# Stratification constrains future heat and carbon uptake in the Southern Ocean between 30°S and 55°S

**DOI:** 10.1038/s41467-022-27979-5

**Published:** 2022-01-17

**Authors:** Timothée Bourgeois, Nadine Goris, Jörg Schwinger, Jerry F. Tjiputra

**Affiliations:** NORCE Norwegian Research Centre, Bjerknes Centre for Climate Research, Bergen, Norway

**Keywords:** Physical oceanography, Marine chemistry, Carbon cycle, Biogeochemistry

## Abstract

The Southern Ocean between 30°S and 55°S is a major sink of excess heat and anthropogenic carbon, but model projections of these sinks remain highly uncertain. Reducing such uncertainties is required to effectively guide the development of climate mitigation policies for meeting the ambitious climate targets of the Paris Agreement. Here, we show that the large spread in the projections of future excess heat uptake efficiency and cumulative anthropogenic carbon uptake in this region are strongly linked to the models’ contemporary stratification. This relationship is robust across two generations of Earth system models and is used to reduce the uncertainty of future estimates of the cumulative anthropogenic carbon uptake by up to 53% and the excess heat uptake efficiency by 28%. Our results highlight that, for this region, an improved representation of stratification in Earth system models is key to constrain future carbon budgets and climate change projections.

## Abstract (web summary)

The heat and carbon uptakes in the Southern Ocean between 30°S and 55°S are critical for mitigating climate change. Here the authors find that projection uncertainties of both uptakes are reduced drastically when using the water column stability as an emergent constraint.

## Introduction

The Southern Ocean is a dynamically complex region. The strong wind-driven Antarctic Circumpolar Current drives a residual overturning circulation, consisting of an upwelling of circumpolar deep waters around the Polar Front, a residual northward transport with gradual water mass transformation to Antarctic Intermediate and Mode Waters (IW and MW), and finally subduction under subtropical waters. The upwelled water mass is cold and undersaturated with respect to anthropogenic carbon, allowing it to efficiently absorb large amounts of atmospheric excess heat and anthropogenic carbon^1–3^. Therefore, the subduction of IW and MW masses, occurring approximately between 30°S and 55°S, provides one of the major gateways carrying anthropogenic carbon (C_ant_) and excess heat (H_excess_) into the interior ocean (e.g. refs. ^4–7^), where they stay isolated from the atmosphere on decadal to millennial timescales^8,9^. For the historical period from 1850 to 2005, it has been estimated that 43% of C_ant_ and 75% of H_excess_ have entered the ocean south of 30°S^10^, although the Southern Ocean accounts for only 30% of the total ocean surface area. Over the same period, the region between 30°S and 55°S is responsible for 27 and 50% of the global ocean C_ant_ and H_excess_ uptake despite covering only 21% of the world ocean, according to the models analysed in this study.

**Fig. 1:**
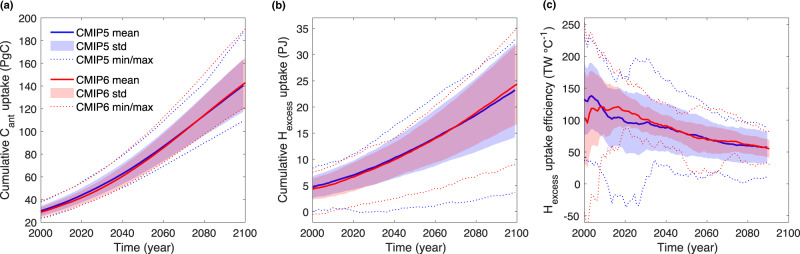
Projections of carbon and heat uptake in the Southern Ocean (30°S–55°S). Southern Ocean (30°S–55°S) **a** cumulative C_ant_ uptake, **b** cumulative H_excess_ uptake and **c** H_excess_ uptake efficiency, i.e. mean excess heat uptake per degree of global warming (Methods) averaged over 17 CMIP5 and 16 CMIP6 models (described in Supplementary Tables 1 and 2) for historical, RCP8.5 (CMIP5) and SSP5-8.5 (CMIP6) experiments. Shaded areas and dotted lines represent one standard deviation (std) and the minimum/maximum (min/max) of the inter-model spread, respectively.

Future projections of C_ant_ and H_excess_ uptake in the region between 30°S and 55°S from the last two generations Earth system models (ESMs) remain uncertain^11,12^ (Fig. 1) because ESMs struggle to capture the complex dynamical and biogeochemical processes in this region^13–15^. Despite improvements in model performance in successive phases of the Coupled Model Intercomparison Project (CMIP), this progress might be too slow to warrant significantly reduced uncertainty of ESM projections within the next decade^16^. Since this is the time horizon for framing climate mitigation policies that allow for meeting stringent climate targets^17,18^, more efforts have to be put into model analysis, i.e., understanding the roots of this uncertainty and reducing uncertainty in key climate metrics such as the projected carbon and heat uptake. The technique of emergent constraints provides a means to constrain a model ensemble through an emergent strong statistical relationship between an observable quantity of current climate and future changes in a variable of interest^19,20^. It has been used to constrain several aspects of the terrestrial^21–23^ and marine^24–26^ carbon cycle.

In this work, we identify a key mechanism that explains the large inter model-uncertainty in future projections of H_excess_ and C_ant_ uptake between 30°S and 55°S across both CMIP5 and CMIP6 ESMs. We focus on the region between 30°S and 55°S since this is the area where intermediate and mode waters are formed and subducted (Methods). We find that the climatological stratification state in this region is tightly related to this subduction and we use this finding to robustly constrain both future H_excess_ and C_ant_ uptake. We note that our definition of C_ant_ and H_excess_ includes changes induced by climate change such as changes in ocean circulation, wind conditions and primary production (Methods).

## Results

### Linking stratification to oceanic C_ant_ and H_excess_ uptake

**Fig. 2:**
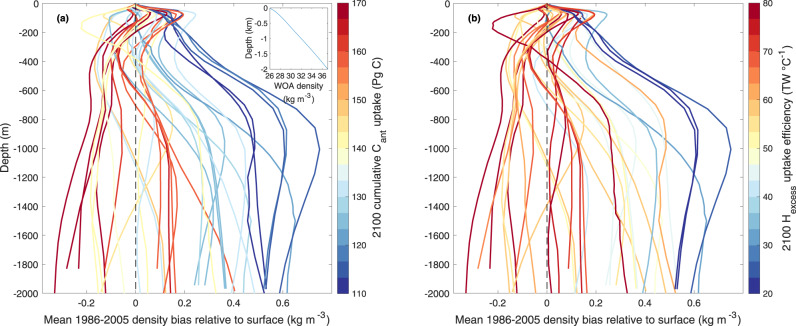
Linkage between contemporary model density bias and projected future ocean carbon and heat uptake. Vertical profiles of Southern Ocean (30°S–55°S) density bias (kg m^−3^) of CMIP5/6 models averaged over 1986–2005 relative to the density of the World Ocean Atlas 2013^54,55^. The surface bias is subtracted from each profile, such that profiles start with a value of zero at the surface. Negative (positive) density bias relative to surface bias indicates weaker- (stronger-) than-observed stratification. Each line shows a single ESM and the colour indicates the magnitude of **a** cumulative C_ant_ uptake and **b** H_excess_ uptake efficiency for the year 2100. The World Ocean Atlas (WOA) density profile is provided as an inset of panel (**a**).

We find that stratification biases in CMIP5 and CMIP6 ESMs in the region between 30°S and 55°S are strongly related to the amount of their future uptake of excess heat per degree of transient global warming (H_excess_ uptake efficiency) and anthropogenic carbon (Fig. 2). Models showing a positive density bias that increases with depth relative to the surface bias (indicating stronger-than-observed stratification) tend to simulate a low uptake of C_ant_ and low H_excess_ uptake efficiency. The opposite is true for models that show an increasingly negative density bias profile relative to their surface bias (indicating weaker-than-observed stratification).

In order to develop an emergent constraint from this apparent relationship, we need to capture the characteristics of the vertical structure of these density profiles in one metric. To this end, we use a stratification index^27^, which is the cumulative sum of density differences with respect to surface density (Methods), here applied over the upper 2000 m of the water column. This depth range has been chosen as it encompasses the MW and IW formation and subduction pathways in CMIP ESMs^7,28^, and modern observational coverage is good, since it is covered by standard ARGO floats.

We identify the core of IW in each ESM by determining the depth of the salinity minimum at 30°S (ref. ^28^), and we find that the stratification index is highly correlated to both (1) the depth at which IW are subducted (R = −0.83) and (2) the subducted volume of IW and overlying MW (R = −0.77, both shown in Supplementary Fig. 1). Therefore, consistent with a previous study^29^, we find that the modelled volume of MW and IW formation is of high importance for determining the efficiency of C_ant_ and H_excess_ sequestration. However, the stratification index has the clear advantage of being straightforward to estimate from model output while the identification of water masses is more challenging and model-dependent^28^.

**Fig. 3:**
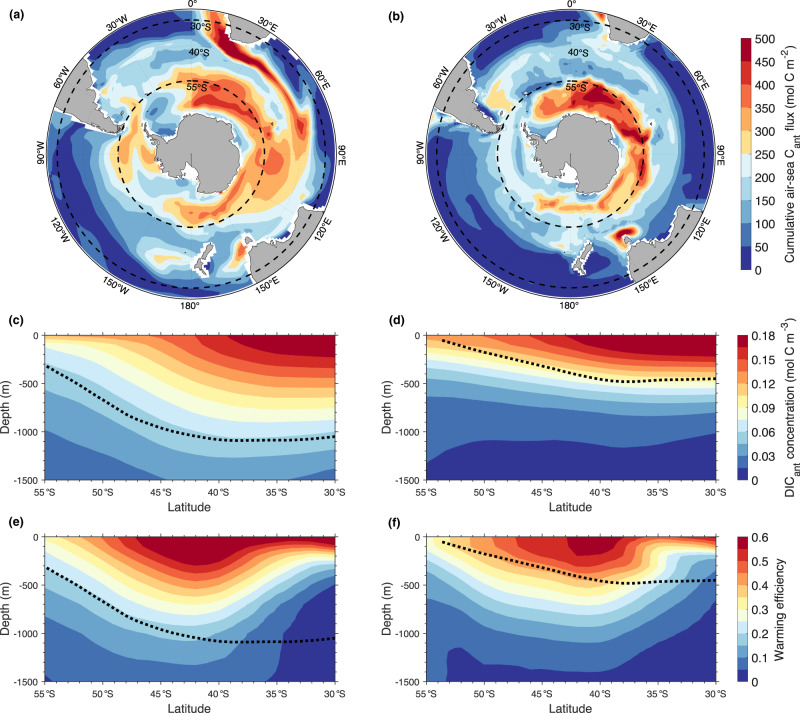
Surface and interior distribution of carbon and heat for models with contrasting stratification states. **a**, **b** Model-mean of the 1850–2100 cumulative C_ant_ uptake for scenario RCP8.5 from **a** the three lowest-stratification-index CMIP5 models (CMCC-CESM, IPSL-CM5A-LR and IPSL-CM5A-MR) and **b** the three highest-stratification-index CMIP5 models (GFDL-ESM2G, HadGEM2-CC and HadGEM2-ES). Black dashed lines show the boundaries of our Southern-Ocean region (30°S–55°S). **c**–**f** Corresponding zonally-averaged transects from the **c**, **e** low- and **d**, **f** high-stratification models for dissolved inorganic C_ant_ concentration and warming efficiency. The latter is defined as the ratio between the transient minus piControl temperature anomaly and the global surface atmospheric warming. The black dotted lines in panels **c**–**f** represent the zonally averaged density isolines crossing the depth of the salinity minimum at 30°S^28^. See Supplementary Fig. 2 for the corresponding CMIP6 models.

We find that ESMs with high-stratification index and correspondingly low C_ant_ uptake typically simulate lower uptake in the region around 55°S, but more importantly, the northward extent of their uptake is much more limited compared to models with low stratification index (Fig. 3). The latter models project accumulated uptake of more than 100 mol C m^−2^ in large regions north of 40°S in Pacific, Indian and Atlantic sectors, where low C_ant_-uptake models show uptake below 50 mol C m^−2^ (see also Supplementary Fig. 2 for a zonal mean view of C_ant_ uptake and Supplementary Fig. 3 for an equivalent to Fig. 3 but for CMIP6 models). The higher (lower) C_ant_ uptake simulated by ESMs with low (high) stratification index is connected to a steeper (shallower) surface-to-depth gradient of the anthropogenic component of dissolved inorganic carbon (DIC_ant_) concentration along the vertical zonal mean section between 30°S and 55°S (Fig. 3c, d). A similar surface-to-depth feature can also be seen in the warming efficiency (Fig. 3e, f).

It is physically plausible for a model that exhibits a stronger stratification than observed to take up less carbon and heat than a model with weaker than observed stratification. However, the fact that present-day stratification is related to projected future uptakes across our model ensemble is not obvious, since stratification is changing with progressing climate change. We find that, in the region between 30°S and 55°S, the projected stratification bias of models relative to each other remains largely unchanged, i.e., a model that simulates a stronger contemporary stratification than the multi-model mean will do so for future time periods, too. The correlation between the mean present-day (1986–2005) stratification index and the mean future (2080–2099) stratification index is 0.91 (Supplementary Fig. 4).

The target variables for our constraints are (i) the cumulative ocean C_ant_ uptake [Pg C] as this minimises interannual and decadal variability (compared to annual carbon fluxes) while preserving trends and (ii) the 20-year average of ocean H_excess_ uptake efficiency [TW °C^−1^] defined as the ratio between ocean H_excess_ uptake rate [TW] and global atmospheric surface warming [°C] (ref. ^30^). The latter choice is motivated by the fact that the simulated atmospheric surface temperature that forces the oceanic heat uptake rates depends on each model’s response to radiative forcing. We, therefore, normalise the excess heat uptake by global surface warming^31–33^. Additional information on the validity of our findings for the H_excess_ uptake rate [TW] without normalisation are presented in the supplement (Supplementary Fig. 5).

In our analysis of H_excess_ uptake efficiency, we merge the CMIP5 and CMIP6 ensembles because both rely on scenarios with the same end of century radiative forcing (RCP8.5 and SSP5-8.5, respectively). Such a merger is not meaningful for the C_ant_ uptake as the CMIP6 SSP5-8.5 scenario reaches considerably higher end-of-century atmospheric CO_2_ concentrations than the CMIP5 RCP8.5 scenario^34^. The radiative forcing due to higher CO_2_ concentrations in SSP5-8.5 is compensated by lower concentrations of other greenhouse gases, mainly methane and nitrous oxide.

### Reducing uncertainties in ocean uptake projections

**Fig. 4:**
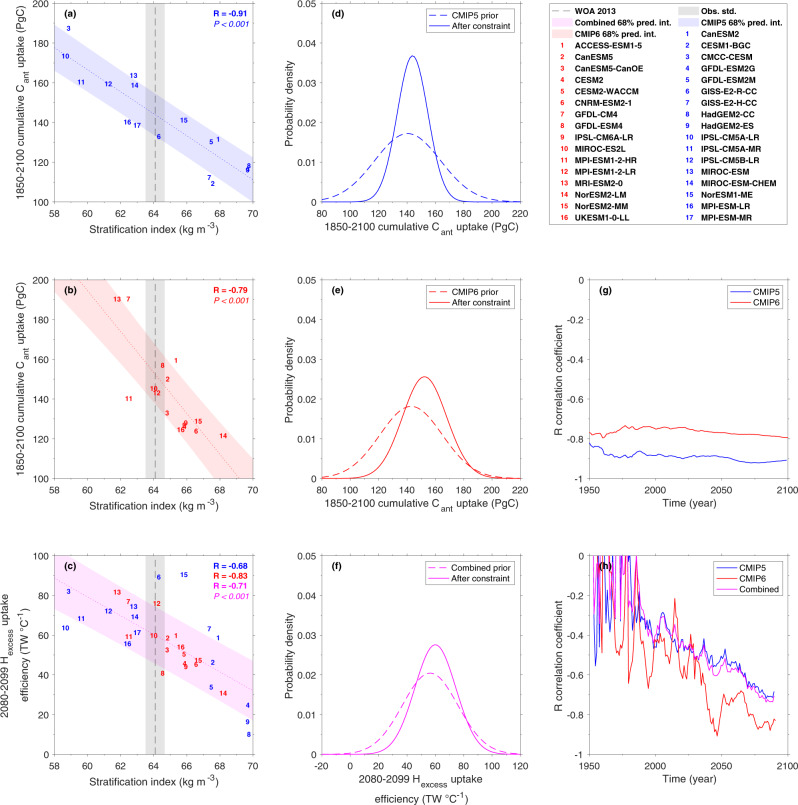
Emergent constraints on the sensitivity of projected carbon and heat uptake to stratification. **a**, **b** cumulative future C_ant_ uptake between 30°S and 55°S and **c** future ocean H_excess_ uptake efficiency and their emergent relation to the contemporary stratification. CMIP5, CMIP6 and combined CMIP5/6 ensembles are denoted in blue, red and magenta, respectively. Emergent constraints include a linear regression fit (dotted line), its 68% prediction interval (abbreviated pred. int., coloured shaded area according to the ensemble), the observational constraint (vertical black dots) and its uncertainty (grey shaded area). All emergent constraints are accompanied by **d**–**f** prior- and after-constraint probability density functions and **g**, **h** correlation time-series obtained by sliding the predictand over time while leaving the predictor fixed.

Significant negative correlations exist between the simulated present-day water-column stratification index and both cumulative C_ant_ uptake and H_excess_ uptake efficiency at the end of the century (Fig. 4a–c). We note that the correlations between the stratification index and H_excess_ are still significant but less robust without normalising the H_excess_ uptake (Supplementary Fig. 5). These correlations indicate that a more stratified ocean absorbs less C_ant_ and H_excess_. For C_ant_ uptake, the high correlation with contemporary stratification is very stable over time (Fig. 4g), whereas for H_excess_ uptake efficiency the correlation is initially low but gets stronger with time (see Discussion) and reaches values of 0.7 (*P* = 0.003) for CMIP5 and 0.8 (*P* < 0.001) for CMIP6 at the end of the century (Fig. 4h). The WOA13-based stratification index and its uncertainty are estimated as 64.08 ± 0.58 kg m^−3^ (Methods). This value is close to the CMIP5 and CMIP6 ensemble mean of 64.72 ± 3.80 kg m^−3^ and 65.02 ± 1.70 kg m^−3^, respectively. However, the model spread around the mean is substantial for both CMIP5 and CMIP6, with CMIP5 having a model uncertainty that is more than twice as large as the one of CMIP6.

Based on the high correlations for both the CMIP5 and CMIP6 ensembles, we apply the emergent constraint approach (Methods) to constrain the uncertainty in future projections of C_ant_ uptake and H_excess_ uptake efficiency. We assume that all models are independent as done in other studies^35^. We note that this is a limitation of our study as some of these ESMs share components and code. Likewise, many CMIP6 models have been developed starting from their predecessor CMIP5 models such that the two ensembles are not entirely independent. An alternative approach could be based on an adaptive model weighting scheme or an ensemble reduction^36,37^, but this is beyond the scope of our study. After applying the observational constraint (Methods), the uncertainties of the cumulative C_ant_ uptake between 30°S and 55°S are considerably reduced by 53 and 32% for CMIP5 and CMIP6, respectively. The associated best estimate of cumulative C_ant_ uptake increases by 3 and 6% for CMIP5 and CMIP6 respectively compared to the prior-constraint estimate (Table 1). Similarly, the after-constraint uncertainty of H_excess_ uptake efficiency for the combined CMIP5/CMIP6 ensemble is strongly reduced by 28% and the associated estimate increases by 7%.

**Table 1:** A prior and revised estimates of future carbon and heat uptake. Prior- and after-constraint estimates and uncertainties (expressed as model mean and standard deviation) of the CMIP5, CMIP6 and combined ensemble in the Southern Ocean (30°S–55°S) of cumulative C_ant_ uptake (Pg C) and H_excess_ uptake efficiency (TW °C^−1^).

Variable	Ensemble	Prior-constraint	After-constraint	Uncertainty change
**Σ** C_ant_ (Pg C)	CMIP5	140 ± 23	144 ± 11	−53%
**Σ** C_ant_ (Pg C)	CMIP6	143 ± 22	152 ± 15	−32%
H_excess_ Eff. (TW °C^−1^)	Combined	56 ± 20	60 ± 14	−28%

Uncertainty changes between prior- and after-constraint are given in %. Abbreviations are included for cumulative (**Σ**) and efficiency (Eff.).

## Discussion

Our emergent constraint identifies a strong link between contemporary stratification in CMIP5/6 models and their ability to continuously take up C_ant_ and H_excess_ under a high-CO_2_ future scenario in the Southern Ocean between 30°S and 55°S. The ESMs’ stratification index correlates strongly with (i) the simulated depth at which the IW (and the overlying MW) are subducted and (ii) the simulated subducted water volume, here loosely referred to as the volume above the IW core (both shown in Supplementary Fig. 1). This suggests that a deeper position of the IW core is accompanied by a larger subduction volume, and hence a more efficient C_ant_ and H_excess_ sequestration in our model ensemble. This importance of the volume of ventilated waters for future C_ant_ uptake in the Southern Ocean has been found in an independent study^29^. We note that the relationship between contemporary stratification and C_ant_ and H_excess_ uptake worsens when extending the region of interest south of 55°S and outside of the area of IW and MW subduction. Here, the C_ant_ and H_excess_ uptake is sensitive to other processes, such as sea-ice dynamics and bottom-water formation at the southward limb of Southern Ocean overturning circulation, and we find no direct link to the contemporary stratification (Supplementary Fig. 6).

It has been shown before that formation of mode and intermediate waters is key for carbon and heat uptake^5,38^, and that this water mass formation appears to be linked to the simulated winter mixed layer depths of CMIP5 models^14^. We find, however, that the relationship between future cumulative C_ant_ and H_excess_ uptake efficiency and mixed layer depth in our region is only weak. There is no significant correlation between annual mean or maximum winter mixed layer depth and carbon and heat uptakes (Supplementary Figs. 7, 8). Deep winter mixing in our region is thought to be the main contributor to carbon and heat subduction, and therefore such relatively low correlations might seem surprising. A previous study^14^ has shown that stratification biases in ESMs contribute to setting the maximum winter mixed layer depth, and this effect is also captured by our constraint. In addition, other processes (such as diapycnal mixing and Ekman pumping) are also important for the eventual subduction of carbon and heat away from the seasonally varying base of the mixed layer^39,40^. A robust relationship can be found when taking the stratification of the upper 2000 m of the water column into account, but we note that our constraint is not sensitive to the exact lower bound of this depth range when it is varied between 1000 and 2000 m. We translate these findings into a physically plausible and robust emergent constraint for future projections of two generations of ESMs. It provides us with the unique possibility to constrain the model uncertainty for two highly important quantities at the same time.

For the CMIP5/6 generation of models, we find that the simulated contemporary stratification between 30°S and 55°S is highly correlated to its projected future values across CMIP5/6 (R correlation of 0.91). Models with a strong stratification store more of the excess heat in the upper ocean, thereby creating stronger stratification changes, while the opposite is true for weakly stratified models^41^. This mechanistic explanation suggests that ESMs that simulate a realistic contemporary density profile are more reliable in simulating future density profiles. The predictor of our emergent constraint, i.e. the contemporary stratification, hence also constrains the future stratification and, as demonstrated here, the future cumulative C_ant_ uptake and the H_excess_ uptake efficiency. A recent study indicates that projected patterns of heat storage are primarily dictated by the preindustrial ocean circulation^42^. Contemporary oceanic storage of anthropogenic carbon and excess heat have distinct patterns^43–45^ and redistributed heat and carbon are projected to have opposing signs, leading to a more horizontal structure of heat storage than seen in the patterns of carbon storage in the Southern Ocean^46^. However, these differences are reduced in future projections of the Southern Ocean as the spatially averaged added heat becomes dominant over the redistribution of heat due to circulation changes^42,46^. This is consistent with our findings, specifically with the initially low but increasing correlation between stratification and excess heat uptake efficiency (Fig. 4e). We note that other quantities like the nutrient cycle and primary productivity are also closely linked to stratification, e.g. it has been shown that CMIP5 models with a stronger bias in contemporary surface stratification tend to predict larger climate-induced declines in surface nutrients and net primary production^47^. Due to the high importance of contemporary stratification biases for future marine projections, it is essential to reduce them.

Our results identify significant stratification biases for most CMIP5/6 models in the areas of MW and IW formation, but also that the representation of stratification in the Southern Ocean between 30°S and 55°S has improved between CMIP5 and CMIP6. In fully coupled ESMs, it remains difficult to identify the ultimate source of biases. The emergent-constraint method is only able to identify systematic biases associated with the variables used in the emergent-constraint relationships. It does not highlight missing processes or dynamical biases common in ESMs which are not directly related to the observable processes or variables used in the constrained process^35,37,48^. As many of the model biases in the Southern Ocean temperature and salinity structure are concentrated in recently ventilated layers or in the deep Atlantic, they appear to stem from inaccuracies in the North Atlantic Deep Water formation regions or in the surface climate over the Southern Ocean^16^. Recently, a strong emergent constraint relationship has been found between surface salinity and cumulative C_ant_ uptake in the Southern Ocean^49^. In combination with our constraint, this indicates that surface salinity is a fundamental player setting the Southern Ocean stratification. Here, the upper ocean properties like salinity are highly sensitive to a multitude of uncertainties in the sea-ice, ocean, and atmosphere components of an ESM, e.g. westerly jet position, Antarctic sea-ice extent and its potential relation to precipitation, clouds, mixing and transport by eddies. It takes a tremendous effort to model or parameterise all these processes in a realistic manner and a significant reduction of bias is not to be expected within this decade^16^. For the ocean, it has been found that eddy-induced diffusion is an important factor in setting the simulated stratification^41^. Hence, a better representation of eddies, be it through increased eddy-resolving resolution^50^ or through improved eddy parameterisations^51^ will very likely contribute to reducing stratification biases in ESMs. Future studies should elucidate processes that could contribute to the bias and large spread in the stratification index simulated across ESMs, for instance, our stratification index could be influenced by the water mass properties of the circumpolar deep water, which is formed in the North Atlantic. A better understanding of the linkage between North Atlantic climate representation and the Southern-Ocean water-mass properties across ESMs could be valuable.

Ensembles of ESMs remain our only tool at hand to investigate the response of the Earth system to future scenarios of anthropogenic forcing. Reducing the large uncertainties arising from, among others, the representation of Southern Ocean dynamics in these models remains a challenge. The identification of emergent constraints, such as the one presented here, are invaluable as they can help to guide model development and, importantly, to speed up the provision of critical knowledge on expected future changes.

## Methods

### CMIP5/6 ensembles

Our ensembles (summarised in Supplementary Tables 1, 2) are based on 17 CMIP5 and 16 CMIP6 ESMs used in the Fifth and Sixth Assessment Report of the Intergovernmental Panel on Climate Change, respectively^52^. INM-CM4 has been excluded from our analysis because the model shows an outlying large density bias for both Intermediate Water (IW) and Mode Water (MW)^28^. We use a single ensemble member (r1i1p1(f1) or equivalent) per model. The selected ESMs provide full periods of the following three standard CMIP5 (CMIP6) experiments: piControl, historical and RCP8.5 (SSP5-8.5). For our study, ocean H_excess_ uptake, ocean C_ant_ uptake and global atmospheric surface warming are calculated using the air-sea heat flux, the air-sea CO_2_ flux and the surface air temperature, respectively. The anthropogenic or excess component is obtained as the difference between the historical or future scenario and the preindustrial control experiments. Thus, C_ant_ and H_excess_ include changes induced by climate change (e.g. changes in ocean circulation, wind conditions, primary production).

### Latitudinal extent of the region considered for the constraint

In our study, we focus on the area where intermediate and mode waters are formed and subducted as these processes are the main drivers of ocean carbon and heat uptake in the Southern Ocean^10,28,38^. This area lies between 30°S and 55°S in all CMIP5 and CMIP6 models. The 30°S is a commonly used northern boundary for the Southern Ocean and its subduction region^5,10,21^. The 55°S southern boundary is chosen to exclude the influence of sea-ice on air-sea fluxes, which would complicate the uptake-stratification relationship (Supplementary Fig. 9). According to the CMIP5 and CMIP6 zonal wind stress distribution^16^, the 55°S southern boundary generally excludes the southward limb of the Southern Ocean overturning circulation, that is not related to the subduction process of interest in this study.

### Density calculations and stratification index

We calculated in situ density (⍴) from each ESM´s potential temperature and practical salinity (after conversion to absolute salinity and conservative temperature) following TEOS-10 standards^53^. Three-dimensional ⍴ fields have been area-weighted and averaged along horizontal surfaces to produce one-dimensional vertical profiles in native (model-dependent) vertical resolution.

We use a Stratification Index (SI) based on ref. ^27^ to characterise the stratification of the water column:

1 $${{{{{\rm{SI}}}}}}=\mathop{\sum }\limits_{i=1}^{10}{\rho }^{{z}_{i}}-{\rho }^{{z}_{0}}$$

where *z*_0_ is the sea surface and *z*_*i*_ = *z*_*i*−1_ + 200 for *i* = 1, …, 10

### Probability density functions for the emergent constraints

The prior probability density functions for cumulative C_ant_ uptake and H_excess_ uptake efficiency assume that all models are equally likely to be correct and lead to a Gaussian distribution, and so is the probability density function of the observational constraint *P*(*x*)^21^. The probability density function of the constrained estimate *P*(*y*) was generated following established methodologies by normalising the product of the conditional probability density function of the emergent relationship *P*(*y│x*) and the probability density function of the observational constraint *P*(*x*):^21,22,25,26^

2 $$P\left(y|x\right)=\frac{1}{\sqrt{2\pi {{\sigma }_{f}}^{2}}}{\exp }\left\{\frac{{(y-f(x))}^{2}}{2{{\sigma }_{f}}^{2}}\right\}$$

where *x* and *y* are the predictor and the predictand, respectively. σ_*f*_ = σ_*f*_(*x*) and is the ‘prediction error’ of the emergent linear regression.

3 $$P\left(y\right)= \int_{-{{\infty }}}^{+{{\infty }}}P\left\{y|x\right\}\,P\left(x\right)\,{dx}$$

### Observational constraint

The World Ocean Atlas 2013 version 2 (WOA13) annual climatology of ⍴ (refs. ^54,55^) is used as an observation-based estimate of the stratification index. The same horizontal area-weighting treatment as for the ESMs is applied to the three-dimensional ⍴ field of WOA13 leading to a finer vertically-resolved (102 levels) one-dimensional vertical profile. ⍴ anomaly profiles comparing the ESMs and WOA13 are computed by vertically interpolating the high-resolution WOA13 ⍴ profile to each coarsely-resolved model levels. The standard deviation of the WOA13 climatological monthly mean ⍴ is used as a proxy for the uncertainty around the climatological mean, as such uncertainty is not provided in the WOA13 database^26^. Standard statistical formulas^56^ for uncertainty propagation are applied for the three-to-one-dimensional reduction.

The SI standard deviation (σ_SI_) of the observational constraint is calculated from the SI formula:

4 $${\sigma }_{{{{{{\rm{SI}}}}}}}=\sqrt{\mathop{\sum }\limits_{i=1}^{10}\overline{\,{\sigma }_{{\rho }^{{z}_{i}}}^{2}}\,+\overline{\,{\sigma }_{{\rho }^{{z}_{0}}}^{2}}}$$

where ${\sigma }_{{\rho }^{{z}_{0}}}$ and ${\sigma }_{{\rho }^{{z}_{i}}}$ are the WOA13 standard deviations.

## Supplementary information

Supplementary Information

## Data Availability

CMIP5 and CMIP6 outputs are available from the Earth System Grid Federation (ESGF) portals (e.g. https://esgf-data.dkrz.de/). The WOA13 density climatology is available from the National Oceanographic Data Center portal (NODC/NOAA) under https://www.nodc.noaa.gov/OC5/woa13/.

## Code availability

The MATLAB environment was used for statistical processing, model analyses and figure creation. The Gibbs-SeaWater (GSW) Oceanographic Toolbox has been used to convert model sea potential temperature and practical salinity to conservative temperature and absolute salinity, and to calculate in situ density (http://www.teos-10.org/software.htm).
