# Supplementary material for: Stratification constrains future heat and carbon uptake in the Southern Ocean between 30°S and 55°S
Source: Nat Commun. 2022 Jan 17;13:340. doi: 10.1038/s41467-022-27979-5 (PMC8764023; doi:10.1038/s41467-022-27979-5)
Supplement: Supplementary file 1 — Supplementary Information [file 41467_2022_27979_MOESM1_ESM.pdf]

Supplementary information:

"Stratification constrains future heat and carbon uptake in the Southern Ocean"

Supplementary Table 1: CMIP5 ensemble

Model name	Modelling center
CanESM2[1]	CCCma
CESM1-BGC[2,3]	NSF-DOE-NCAR
CMCC-CESM[4,5]	CMCC
GFDL-ESM2G[6]	NOAA GFDL
GFDL-ESM2M[6]	NOAA GFDL
GISS-E2-R-CC[7]	NASA GISS
GISS-E2-H-CC[7]	NASA GISS
HadGEM2-CC[8]	MOHC
HadGEM2-ES[8]	MOHC and INPE
IPSL-CM5A-LR[9]	IPSL
IPSL-CM5A-MR[9]	IPSL
IPSL-CM5B-LR[9]	IPSL
MIROC-ESM[10]	MIROC
MIROC-ESM-CHEM[10]	MIROC
NorESM1-ME[11,12]	NCC
MPI-ESM-LR[13]	MPI-M
MPI-ESM-MR[13]	MPI-M

Supplementary Table 2: CMIP6 ensemble

Model name	Modelling center
ACCESS-ESM1-5[14]	CSIRO
CanESM5[15]	CCCma
CanESM5-CanOE[15]	CCCma
CESM2[16]	NCAR
CESM2-WACCM[16]	NCAR
CNRM-ESM2-1[17]	CNRM-CERFACS
GFDL-CM4[18,19]	NOAA-GFDL
GFDL-ESM4[20,21]	NOAA-GFDL
IPSL-CM6A-LR[22]	IPSL
MIROC-ES2L[23]	MIROC
MPI-ESM1-2-LR[24,25]	MPI-M
MPI-ESM1-2-HR[24,25]	MPI-M
MRI-ESM2-0[26]	MRI
NorESM2-LM[27,28]	NCC
NorESM2-MM[27,28]	NCC
UKESM1-0-LL[29]	MOHC

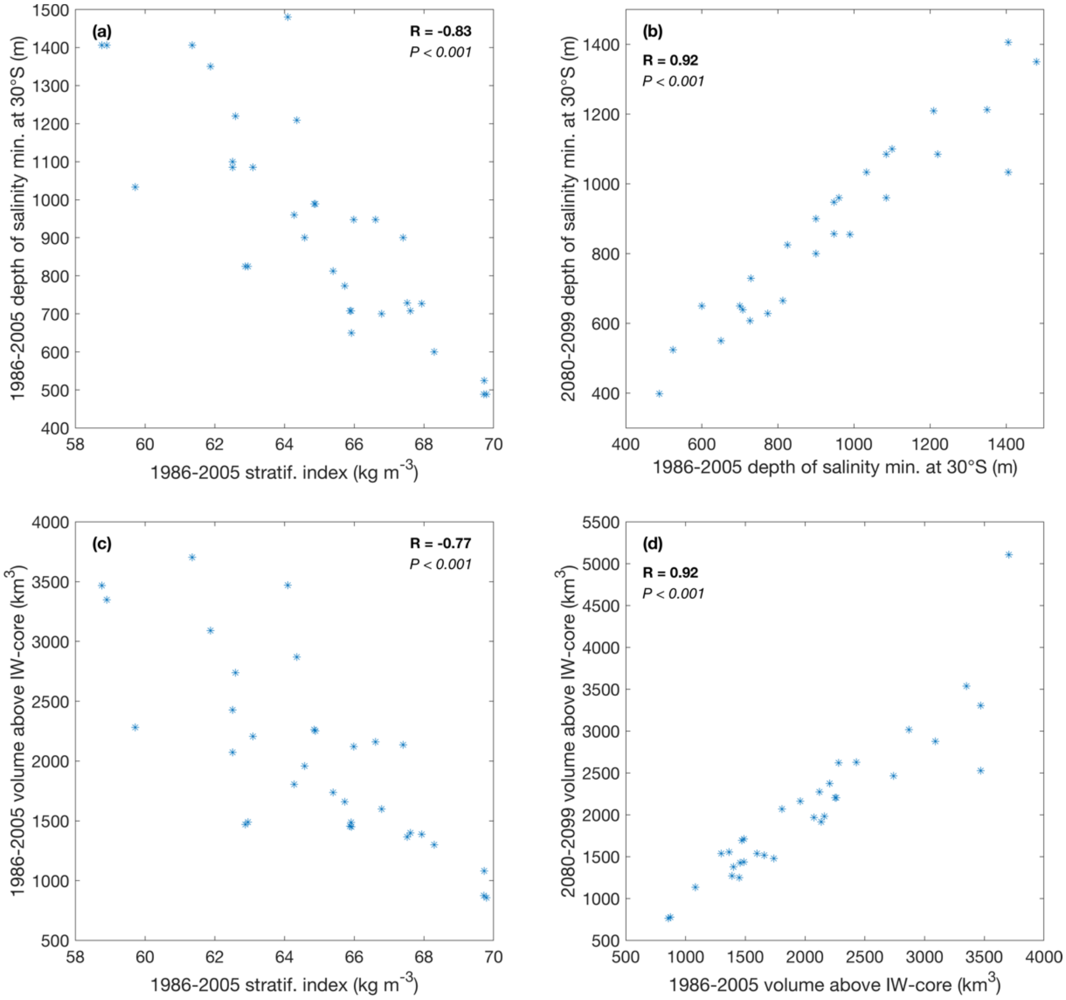

Supplementary Figure 1: Relationship between the 1986-2005 stratification index and (a) the 1986-2005 depth of the zonal average salinity minimum at 30°S and (c) the 1986-2005 volume above the intermediate-water core (IW-core). (b,d) Relationship between contemporary (1986-2005) and future (2080-2099) depth of the salinity minimum at 30°S and the volume of water above the IW-core. The volume above IW-core is defined as the volume between 30°S and 55°S and between the surface and the longitudinally averaged density isoline crossing the depth of salinity minimum at 30°S (ref. [30]). The 1986-2005 and 2080-2099 periods are from the historical and RCP8.5/SSP5-8.5 experiments, respectively. Correlations are given at the top of each panel.

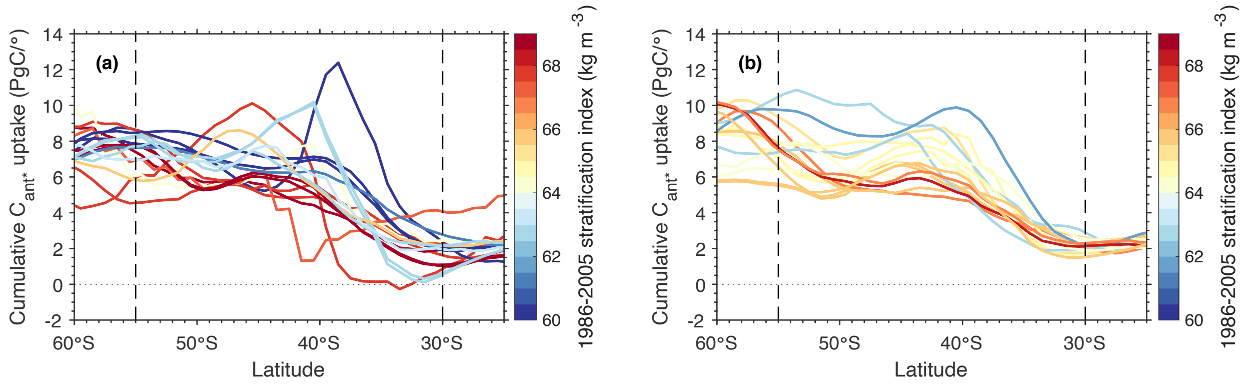

Supplementary Figure 2: Longitudinally averaged zonal 1850-2100 cumulative C_{ant} uptake for the (left) CMIP5 and (right) CMIP6 ensemble. The values are per degree of latitude using historical and RCP8.5/SSP5-8.5 experiments. Each line shows a single ESM and the colour depict its stratification index for the period 1986-2005. Vertical black dashed lines depict the regional boundaries of our Southern-Ocean subduction region.

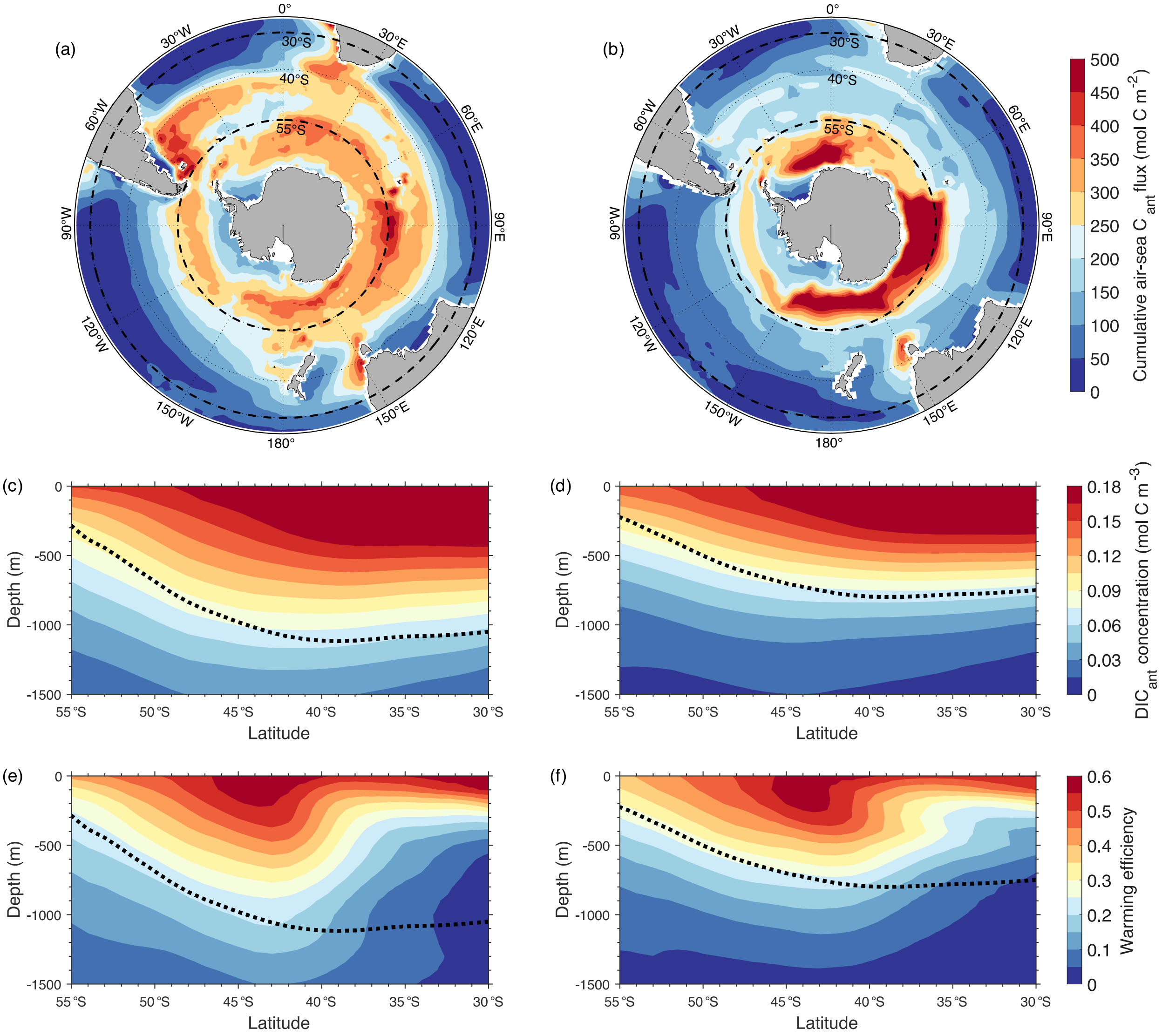

Supplementary Figure 3: Same as Article Figure 3 for CMIP6 models with **(a, c, e)** the low-stratification models (GFDL-CM4, MPI-ESM1-2-HR and MRI-ESM2-0) and **(b, d, f)** the high-stratification models (CNRM-ESM2-1, NorESM2-LM and NorESM2-MM).

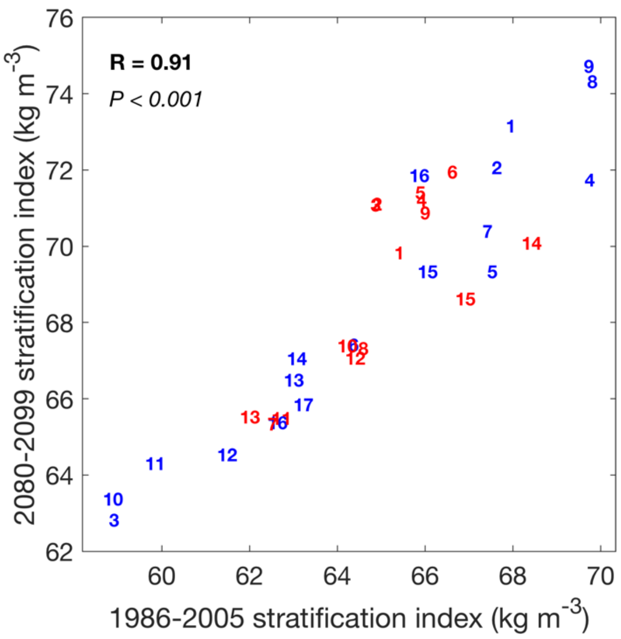

Supplementary Figure 4: CMIP5 and CMIP6 present (Historical 1986-2005) versus future (RCP8.5 / SSP5-8.5 2080-2099) stratification index. See Article Figure 4 for the legend.

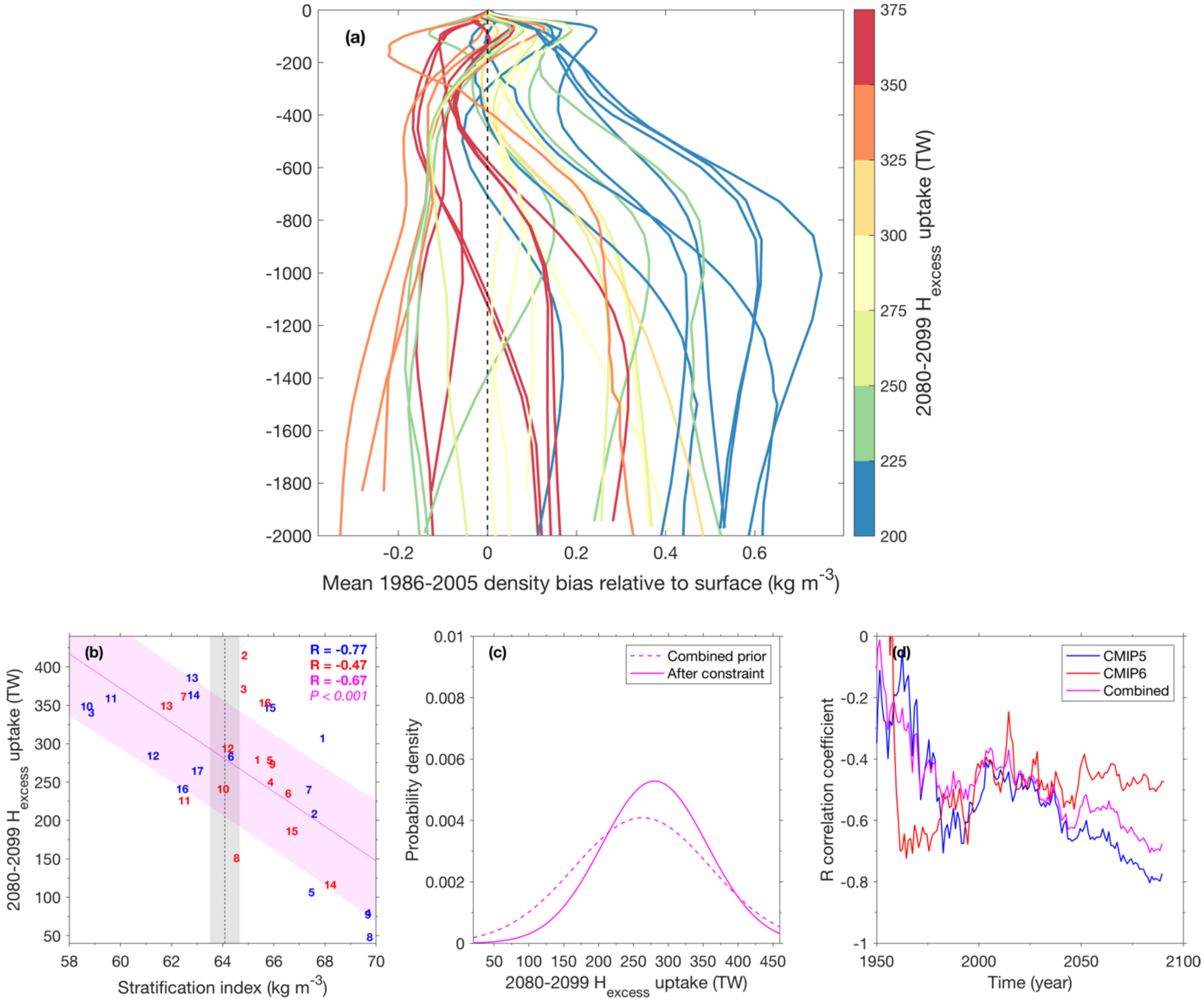

Supplementary Figure 5: Equivalent of (a) Article Figure 2 and (b-d) Article Figure 4 using 2080-2099 H_{excess} uptake.

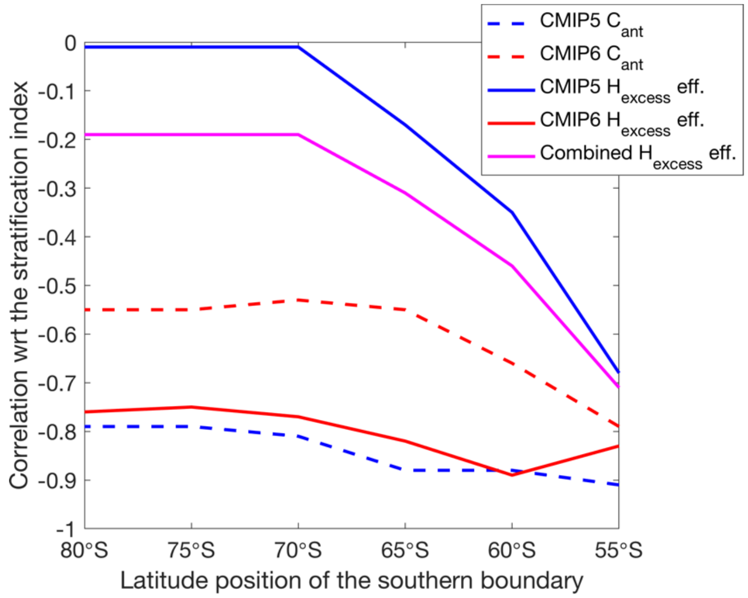

Supplementary Figure 6: Sensitivity analysis of the correlation in the emergent constraint to the position of the southern boundary. Each line represents the correlation coefficient of the emergent constraint as also shown in Figure 4 but using a fixed 30°S northern boundary and a southern boundary moving southwards at 5° intervals.

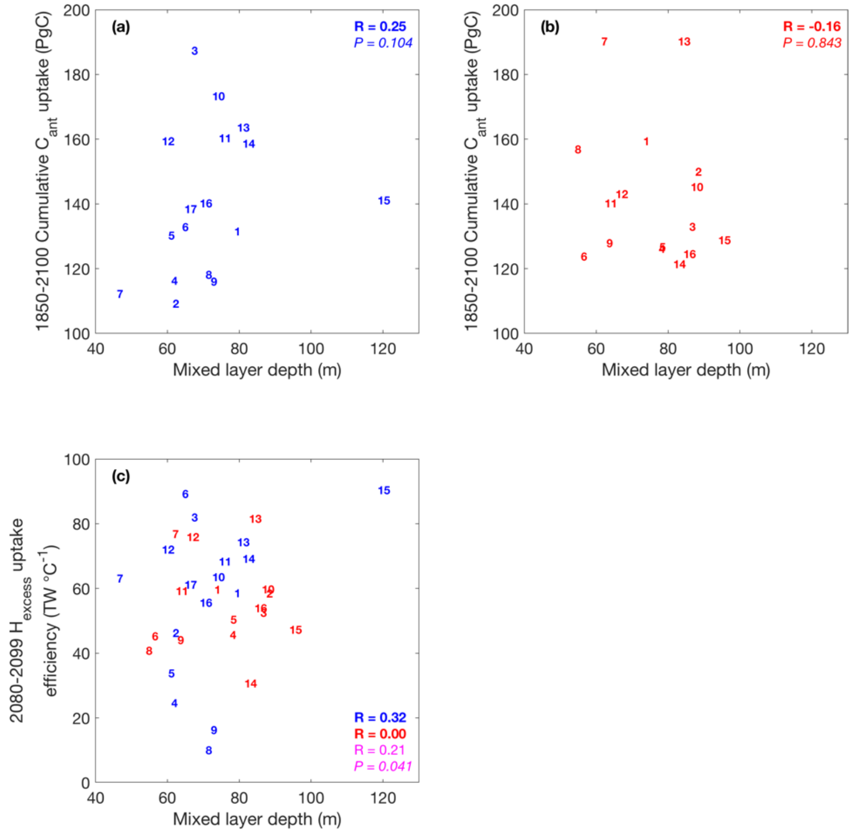

Supplementary Figure 7: Same as Article Figure 4 (a-c) but using contemporary annual mean mixed layer depth. The mixed layer depth is defined using a surface-density difference criterion of

0.03 kg m^{-3} (ref. [31]).

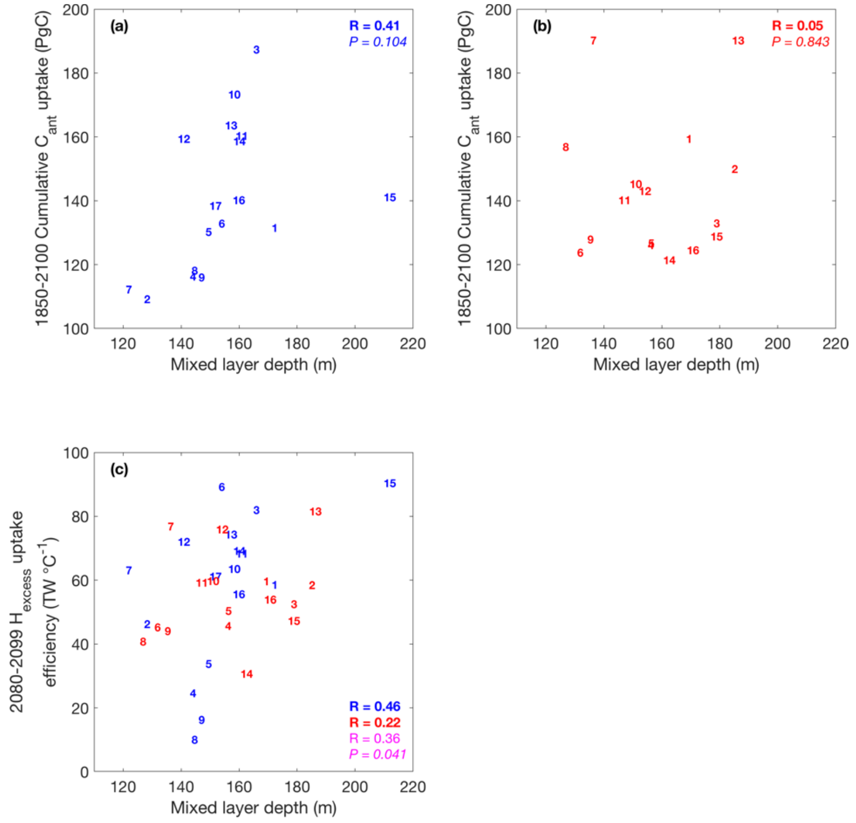

Supplementary Figure 8: Same as Article Figure 4 (a-c) but using contemporary mean winter mixed layer depth, defined as the average of monthly maximum mixed layer depths over 20 years.

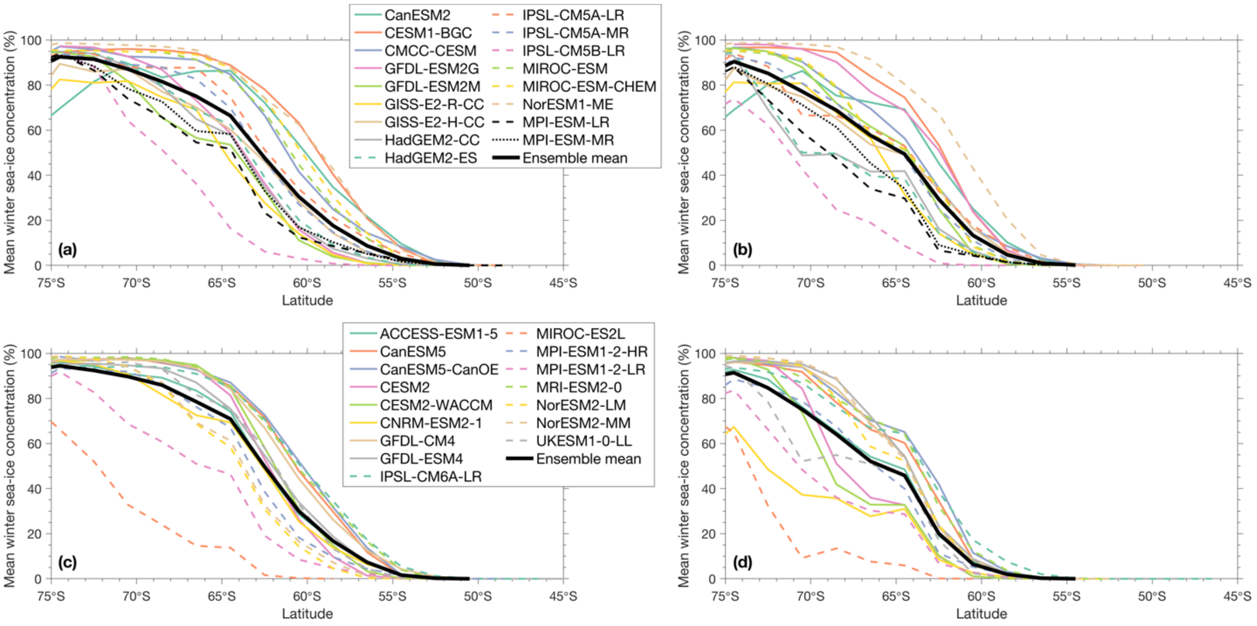

Supplementary Figure 9: Mean longitudinally-averaged winter (July-August-September) sea-ice concentration for **(a, b)** CMIP5 and **(c, d)** CMIP6 from **(a, c)** the historical 1986-2005 period and **(b, d)** the RCP8.5 / SSP5-8.5 2080-2099 projections.

Supplementary References

1. Flato, G. M. et al. The Canadian Centre for Climate Modelling and Analysis global coupled model and its climate. *Clim. Dyn.*, **16,** 451–467, https://doi.org/10.1007/s003820050339 (2000).

2. Gent, P. R. et al. The Community Climate System Model Version 4. *J. Climate*, **24,** 4973–4991, https://doi.org/10.1175/2011JCLI4083.1 (2011).

3. Lindsay, K. et al. Preindustrial-Control and Twentieth-Century Carbon Cycle Experiments with the Earth System Model CESM1(BGC). *J. Climate*, **27,** 8981–9005, https://doi.org/10.1175/JCLI-D-12-00565.1 (2014).

4. Vichi, M. et al. Global and regional ocean carbon uptake and climate change: sensitivity to a substantial mitigation scenario. *Clim. Dyn.*, **37,** 1929–1947, https://doi.org/10.1007/s00382-011-1079-0 (2011).

5. Cagnazzo, C. et al. Role of stratospheric dynamics in the ozone–carbon connection in the Southern Hemisphere. *Clim. Dyn.*, **41,** 3039–3054, https://doi.org/10.1007/s00382-013-1745-5 (2013).

6. Dunne, J. P. et al. GFDL's ESM2 Global Coupled Climate–Carbon Earth System Models. Part I: Physical Formulation and Baseline Simulation Characteristics. *J. Climate*, **25,** 6646–6665, https://doi.org/10.1175/JCLI-D-11-00560.1 (2012).

7. Schmidt, G. A. et al. Configuration and assessment of the GISS ModelE2 contributions to the CMIP5 archive. *J. Adv. Model. Earth Syst.*, **6,** 141–184, https://doi.org/10.1002/2013MS000265 (2014).

8. Collins, W. J. et al. Development and evaluation of an Earth-System model – HadGEM2. *Geosci. Model Dev.*, **4,** 1051–1075, https://doi.org/10.5194/gmd-4-1051-2011 (2011).

9. Dufresne, J.-L. et al. Climate change projections using the IPSL-CM5 Earth System Model: from CMIP3 to CMIP5. *Clim. Dyn.*, **40,** 2123–2165, https://doi.org/10.1007/s00382-012-1636-1 (2013).

10. Watanabe, S. et al. MIROC-ESM 2010: model description and basic results of CMIP5-20c3m experiments. *Geosci. Model Dev.*, **4,** 845–872, https://doi.org/10.5194/gmd-4-845-2011 (2011).

11. Bentsen, M. et al. The Norwegian Earth System Model, NorESM1-M – Part 1: Description and basic evaluation of the physical climate. *Geosci. Model Dev.*, **6,** 687–720, https://doi.org/10.5194/gmd-6-687-2013 (2013).

12. Tjiputra, J. F. et al. Evaluation of the carbon cycle components in the Norwegian Earth System Model (NorESM), *Geosci. Model Dev.*, **6,** 301–325, https://doi.org/10.5194/gmd-6-301-2013, (2013).

13. Giorgetta, M. A. et al. Climate and carbon cycle changes from 1850 to 2100 in MPI-ESM simulations for the Coupled Model Intercomparison Project phase 5. *J. Adv. Model. Earth Syst.*, **5,** 572–597, https://doi.org/10.1002/jame.20038 (2013).

14. Ziehn, T. et al. The Australian Earth System Model: ACCESS-ESM1.5. *J. So. Hemisph. Earth*, https://doi.org/10.1071/ES19035 (2020).

15. Swart et al. The Canadian Earth System Model version 5 (CanESM5.0.3). *Geosci. Model Dev.*, **12,** 4823–4873, https://doi.org/10.5194/gmd-12-4823-2019 (2019).

16. Danabasoglu et al. The Community Earth System Model Version 2 (CESM2). *J. Adv. Model. Earth Syst.*, **12,** 1–35, https://doi.org/10.1029/2019MS001916 (2020).

17. Séférian, R. et al. Evaluation of CNRM Earth System Model, CNRM-ESM2-1: Role of Earth System Processes in Present-Day and Future Climate. *J. Adv. Model. Earth Syst.*, **11,** 4182–4227, https://doi.org/10.1029/2019MS001791 (2019).

18. Held, I. M. et al. Structure and Performance of GFDL's CM4.0 Climate Model. *J. Adv. Model. Earth Syst.*, **11,** 3691–3727, https://doi.org/10.1029/2019MS001829 (2019).

19. Dunne, J. P. et al. Simple Global Ocean Biogeochemistry With Light, Iron, Nutrients and Gas Version 2 (BLINGv2): Model Description and Simulation Characteristics in GFDL's CM4.0. *J. Adv. Model. Earth Syst.*, **12,** https://doi.org/10.1029/2019MS002008 (2020).

20. Dunne, J. P. et al. The GFDL Earth System Model version 4.1 (GFDL-ESM 4.1): Overall coupled model description and simulation characteristics. *J. Adv. Model. Earth Syst.*, **12,** 0–3, https://doi.org/10.1029/2019MS002015 (2020).

21. Stock et al. Ocean Biogeochemistry in GFDL's Earth System Model 4.1 and its Response to Increasing Atmospheric CO 2 . *J. Adv. Model. Earth Syst.*, **12,** https://doi.org/10.1029/2019ms002043 (2020).

22. Boucher, O. et al. Presentation and Evaluation of the IPSL-CM6A-LR Climate Model. *J. Adv. Model. Earth Syst.*, **12,** 1–52, https://doi.org/10.1029/2019MS002010 (2020).

23. Hajima, T. et al. Development of the MIROC-ES2L Earth system model and the evaluation of biogeochemical processes and feedbacks. *Geosci. Model Dev.*, **13,** 2197–2244, https://doi.org/10.5194/gmd-13-2197-2020 (2020).

24. Müller, W. A. et al. A Higher-resolution Version of the Max Planck Institute Earth System Model (MPI-ESM1.2-HR). *J. Adv. Model. Earth Syst.*, **10,** 1383–1413, https://doi.org/10.1029/2017MS001217 (2018).

25. Mauritsen, T. et al. Developments in the MPI-M Earth System Model version 1.2 (MPI-ESM1.2) and Its Response to Increasing CO2. *J. Adv. Model. Earth Syst.*, **11,** 998–1038, https://doi.org/10.1029/2018MS001400 (2019).

26. Yukimoto, S. et al. The Meteorological Research Institute Earth System Model Version 2.0, MRI-ESM2.0: Description and Basic Evaluation of the Physical Component. *J. Meteorol. Soc. Japan Ser. II*, **97,** 931–965, https://doi.org/10.2151/jmsj.2019-051 (2019).

27. Seland, Ø. et al. The Norwegian Earth System Model, NorESM2 – Evaluation of the CMIP6 DECK and historical simulations, *Geosci. Model Dev. Discuss.*, https://doi.org/10.5194/gmd-2019-378 (2020).

28. Tjiputra, J. F. et al. Ocean biogeochemistry in the Norwegian Earth System Model version 2 (NorESM2). *Geosci. Model Dev.*, **13,** 2393–2431, https://doi.org/10.5194/gmd-13-2393-2020 (2020).

29. Sellar, A. A. et al. UKESM1: Description and Evaluation of the U.K. Earth System Model. *J. Adv. Model. Earth Syst.*, **11,** 4513–4558, https://doi.org/10.1029/2019MS001739 (2019).

30. Sallée, J. B. et al. Assessment of Southern Ocean water mass circulation and characteristics in CMIP5 models: Historical bias and forcing response. *J. Geophys. Res. Ocean.*, **118,** 1830–1844, https://doi.org/10.1002/jgrc.20135 (2013).

31. de Boyer Montégut, C., Madec G., Fischer A. S., Lazar A., & Ludicone D. Mixed layer depth over the global ocean: An examination of profile data and a profile-based climatology, *J. Geophys. Res.*, **109,** C12003, https://doi.org/10.1029/2004JC002378 (2004).
